# Imaging of Musculoskeletal Bacterial Infections by [^124^I]FIAU-PET/CT

**DOI:** 10.1371/journal.pone.0001007

**Published:** 2007-10-10

**Authors:** Luis A. Diaz, Catherine A. Foss, Katherine Thornton, Sridhar Nimmagadda, Christopher J. Endres, Ovsev Uzuner, Thorsten M. Seyler, Slif D. Ulrich, Janet Conway, Chetan Bettegowda, Nishant Agrawal, Ian Cheong, Xiaosong Zhang, Paul W. Ladenson, Barry N. Vogelstein, Michael A. Mont, Shibin Zhou, Kenneth W. Kinzler, Bert Vogelstein, Martin G. Pomper

**Affiliations:** 1 The Howard Hughes Medical Institute, The Ludwig Center for Cancer Genetics and Therapeutics at Johns Hopkins, Baltimore, Maryland, United States of America; 2 Russell H. Morgan Department of Radiology and Radiological Sciences, Johns Hopkins University, Baltimore, Maryland, United States of America; 3 Center for Joint Preservation and Reconstruction, Rubin Institute for Advanced Orthopedics, Sinai Hospital of Baltimore, Baltimore, Maryland, United State of America; 4 Division of Endocrinology and Metabolism, Department of Medicine, Johns Hopkins University School of Medicine, Baltimore, Maryland, United States of America; 5 The Sidney Kimmel Comprehensive Cancer Center at Johns Hopkins, Baltimore, Maryland, United States of America; Brighton and Sussex Medical School, United Kingdom

## Abstract

**Background:**

Traditional imaging techniques for the localization and monitoring of bacterial infections, although reasonably sensitive, suffer from a lack of specificity. This is particularly true for musculoskeletal infections. Bacteria possess a thymidine kinase (TK) whose substrate specificity is distinct from that of the major human TK. The substrate specificity difference has been exploited to develop a new imaging technique that can detect the presence of viable bacteria.

**Methodology/Principal Findings:**

Eight subjects with suspected musculoskeletal infections and one healthy control were studied by a combination of [^124^I]FIAU-positron emission tomography and CT ([^124^I]FIAU-PET/CT). All patients with proven musculoskeletal infections demonstrated positive [^124^I]FIAU-PET/CT signals in the sites of concern at two hours after radiopharmaceutical administration. No adverse reactions with FIAU were observed.

**Conclusions/Significance:**

[^124^I]FIAU-PET/CT is a promising new method for imaging bacterial infections.

## Introduction

Conventional anatomic imaging techniques such as magnetic resonance imaging (MRI) and computed tomography (CT) are incapable of reliably distinguishing infection from sterile inflammation. Previous radiopharmaceutical-based imaging techniques for infection are indirect, for example by relying on the accumulation of ^99m^Tc- or ^111^In-tagged leukocytes at infectious sites rather than on detection of the infectious agent *per se.* [1], [2] Recently, the ability to image bacterial infection directly with radiolabeled antibiotics and peptides directed to specific organisms has been studied, but clinical use has been limited, in part because of suboptimal specificity or sensitivity.[3]–[6]

We were initially interested in developing an imaging agent for *in vivo* monitoring of a live bacterial-based cancer therapeutic agent named *Clostridium novyi-NT* (*C. novyi-NT*).[7]–[9] During our investigations, we found that 1-(2′-deoxy-2′-fluoro-β-D-arabinofuranosyl)-5-iodouracil (FIAU) was a substrate for the native thymidine kinase (TK) from a wide variety of bacteria, including *C. novyi-NT*. When [^125^I]FIAU (radiolabeled, or rFIAU) was used as an imaging tracer, it was possible to visually localize a variety of suppurative bacterial infections.[10]

We speculated that radiolabeled analogs of FIAU could also be used to image active bacterial infection in humans. Some of the most challenging types of infections are those involving the musculoskeletal system. These infections can be particularly difficult to diagnose because non-specific symptoms, such as pain, are frequently related to arthritis, trauma, recent surgery, or aseptic loosening of a prosthetic joint.[11] Standard radiographs are of limited value for diagnosis because of the paucity of reliable diagnostic features. Magnetic resonance imaging is not widely used because prostheses or fixation interferes with the quality of the study and the signals are non-specific. Various radionuclide scans have not always demonstrated sufficient sensitivity or specificity for musculoskeletal infections.[12] For example, according to one meta-analysis of imaging of potentially infected feet in 2,889 diabetic patients from 50 studies, ^99m^Tc-tagged leukocytes demonstrated a sensitivity of 86% and specificity of only 84%.[13] Positron emission tomography with FDG-PET has also been used.[14] The test relies on the detection of inflammatory cells with an increased glucose uptake in areas of infection. Various authors have demonstrated accuracy ranging from 47% to 95%.[15]–[17] Thus, even after a patient undergoes several imaging and laboratory evaluations, definitive evidence to rule in or out an infection is often elusive.

In the current study, we used a combination of [^124^I]FIAU-positron emission tomography and CT ([^124^I]FIAU-PET/CT) to test a small number of patients with suspected musculoskeletal infections. We found that [^124^I]FIAU successfully detected bacterial lesions in all eight patients who were subsequently shown to have clinically significant musculoskeletal bacterial infections.

## Methods

### Patients

Eight subjects with proven or suspected musculoskeletal infections and one healthy control from Sinai Hospital (Baltimore, Maryland) or The Johns Hopkins Medical Institutions (Baltimore, Maryland) were enrolled based on predefined eligibility criteria. Both institutions are teaching and tertiary care hospitals and are major referral sites for patients with bone, joint and prosthetic infections. This study was approved by the Institutional Review Boards (IRBs) of Johns Hopkins and Sinai Hospitals. All subjects were enrolled with signed written informed consent in accordance with the regulations of both IRBs. Case histories and tables have excluded non-pertinent data (i.e. age and gender) of individual patients to protect the identity of our subjects. Additional demographic data is available upon request.

### Study Procedure

All patients underwent a thorough screening program for the presence of infectious symptoms including a review of the medical history, physical findings, results of blood tests, plain-film radiography and [^99m^Tc]MDP bone scan (when clinically indicated). In summary, patients were eligible if they were 18 years or older with a high suspicion of bacterial cellulitis or infections of the musculoskeletal system. Patients with suspected infections of the gastrointestinal tract were excluded as were those with prior thyroid abnormalities, transplanted solid organs, liver disease, or human immunodeficiency virus (HIV) infection. High doses of FIAU administered for several weeks have been shown to induce lactic acidosis and liver failure. To avoid a possible deleterious drug interaction, we therefore excluded patients on medications associated with lactic acidosis (e.g., Metformin).

Patients were intravenously administered 74 MBq (2 mCi) of [^124^I]FIAU (SA 8,596-13,979 Ci/mmol, avg. 11,219±1,949 Ci/mmol ) and were imaged by PET/CT without intravenous (IV) or oral contrast at two hours after injection. Four patients were also imaged at 24 hours post injection (indicated in the text). Six of eight patients underwent surgery for management of possible musculoskeletal infections and tissues removed at surgery provided material for cultures upon which their diagnoses were based. Albumin, total bilirubin, alkaline phosphatase, total protein, alanine aminotranferease, aspartate aminotransferase, and thyroid stimulating hormone levels, and prothrombin time were obtained just prior to and two weeks after [^124^I]FIAU injection.

### Thyroid Protection

To limit radiation exposure of iodine-concentrating thyroid tissue that could result from the formation of free [^124^I]iodide from the metabolism of [^124^I]FIAU, patients were given potassium iodide tablets, 130 mg orally once a day, beginning 1 hour before [^124^I]FIAU administration and for a total of 8 doses.

### [^124^I]FIAU synthesis, purification and quality control

The synthesis of [^124^I]FIAU was adapted from Jacobs *et al*.[18] Radiosynthesis of [^124^I]FIAU according to good manufacturing practice (GMP) was preceded by the development of standard operating procedures (SOPs). The GMP protocol was validated through three successful radiosyntheses, each of which conformed to all stipulations of the SOP. Briefly, 40 µg of 1-(2′-fluoro-2′-deoxy-1-β-D-arabinofuranosyl)-uracil (FAU) precursor (ABX, Radeberg, Germany) was added to a 0.3 mL glass v-vial containing a Teflon-lined cap. Between 10 and 20 mCi (370–740 MBq) of [^124^I]NaI (Eastern Isotopes, Sterling, VA) in 30 µL of 0.02 *M* NaOH was then added to the vial, to which was added 100 µL of 2 *M* HNO_3_. The cap was sealed tightly and the reaction was heated to 115°C for 45 min. Following quenching with 100 µL of 0.1 *M* NH_4_OH, [^124^I]FIAU was purified by HPLC using an Alltech Absorbosil RP C_18_ (250×4.6 mm) column eluted with 10% ethanol in 50 m*M* NaH_2_PO_4_, pH 5.3, at a flow rate of 1 mL/min. The absorbance at 254 nm and the gamma radiation were simultaneously monitored and recorded. The collected product was diluted with four volumes of 0.9% NaCl, passed through a 0.22 µm syringe filter and collected into a sterile, pyrogen-free evacuated vial. Three small aliquots of the radiopharmaceutical solution were then taken to assess the radiochemical purity (avg. 99.2±1.3%), radiochemical yield (avg. 45.6±19.2%), specific radioactivity (8,596–13,979 Ci/mmol, avg. 11,219±1,949 Ci/mmol), chemical purity (avg. 0.39±0.35 µg/mL of FAU and 0.090±0.004 µg/mL of FIAU) and chemical identity (via HPLC co-injection with an FIAU standard) (**figure S1**).

Microbial sterility was assessed by direct inoculation of 100 µL of radiopharmaceutical solution into Trypticase Soy Broth and Fluid Thioglycolate Medium vials (Becton Dickenson), followed by aerobic incubation at either 22.5°C or 32.5°C for two weeks. The presence of pyrogens was assessed with a Bacterial Endotoxin Test (Charles River, Wilmington, MA). No pyrogens were detected in any of the radiopharmaceutical doses (≤16 USP Endotoxin Unit/mL).

### PET/CT Imaging and Analysis

Patients received an intravenous injection of 74 MBq (2 mCi) of [^124^I]FIAU. Whole body PET/CT images were acquired at 2 hours and 24 hours (where indicated) post radiotracer administration using a Discovery LS instrument (General Electric Medical Systems). The Discovery LS allows simultaneous acquisition of 35 transaxial images with a slice thickness of 4.25 mm per bed position for the PET images. Typically, six or more bed positions are used for a whole-body study. Transaxial image resolution is approximately 4.5 mm in full width at half maximum. The field of view and pixel size of the reconstructed PET images are 50 cm and 3.91 mm, respectively. This imaging device also allows multi-detector-row helical CT. Whole-body CT images were acquired over 37 seconds. The technical parameters for the CT portion of the examination were as follows: a detector-row configuration of 4×5 mm, a pitch of 6:1 (high-speed mode), a gantry rotation time of 0.8 seconds, a table speed of 30 mm per gantry rotation, 140 kVp, and 40–120 mA (depending on body weight). A whole-body emission PET scan for the same length of coverage was performed, with a 5-min acquisition per bed position. Attenuation-corrected PET images were reconstructed with an iterative reconstruction ordered-subset expectation maximization (OS-EM) algorithm. The 5-mm-thick transaxial CT images were reconstructed at 4.25-mm intervals for fusion with the transaxial PET images. CT, PET, and fused PET/CT images were then generated on a computer workstation. DICOM images were imported into Analyze 7.0 (Mayo Clinic Foundation, Rochester, MN). Regions of interest were drawn manually on 2–4 slices and the activity concentration was determined. Standardized uptake values (SUV) were normalized to lean body mass using a formula that accounts for age, height, and body weight.[19]

## Results

### Imaging of Infections

Only subjects with symptoms suggestive of active musculoskeletal infections were recruited into this study. No patient had received antibiotics within the three weeks prior to imaging. A brief clinical description of each patient and results of the [^124^I]FIAU scan are summarized in **table 1** and discussed in detail below. The first four patients were scanned by PET/CT at 2 and 24 hours after injection of [^124^I]FIAU and showed similar results at both time points. Therefore, all subsequent patients were only scanned at 2 hours. All subjects with confirmed infections had positive [^124^I]FIAU uptake within the anatomical site of interest. The presence of infection was confirmed by intraoperative surgical histological or culture data from the affected tissues. The control subject and patient # 2 had negative scans and neither had clinical evidence of infection at the time of imaging.

**Table 1:** Patient Characteristics

#	Site of Concern	Pre-surgical Diagnosis	Post-Surgical Diagnosis	Culture Results	CRP† (mg/dL)	rFIAU Scan	
						2 hour	24 hour
1	Right knee	Septic arthritis	Septic Arthritis	MRSA	55.0	Positive	Positive
2	Right hip	Septic arthritis	No Infection*	ND	8.9	Negative	Negative
3	Left hip	Septic prosthesis	Septic prosthesis	MRSA	80.1	Positive	Positive
4	Right knee	Septic arthritis	Septic Arthritis	MRSA	51.0	Positive	Positive
5	Right tibia	Osteomyelitis	Osteomyelitis	*Proteus mirabilis, gram positive cocci*	9.5	Positive	ND
6	Left tibia	Osteomyelitis	Osteomyelitis	No growth	5.3	Positive	ND
7	Left lower extremity	Cellulitis	No Surgery	*E. faecalis, MSSA, psuedomonas sp., E.coli and Proteus sp.*	ND	Positive	ND
8	Left knee	Septic arthritis	Necrotizing septic arthritis	MRSA	150.0	Positive	ND
9	Control	Control	Control	ND	ND	Negative	ND

† Normal C-reactive protein (CRP) levels: <0.5 mg/dL

* Clinical symptoms concerning for infection resolved without antibiotics or surgery-no infection was diagnosed

MRSA-Methicillin-resistant *Staphylococcus aureus*, MSSA-Methicillin-sensitive *Staphylococcus aureus*

ND-Not Done

**Figure 1:**
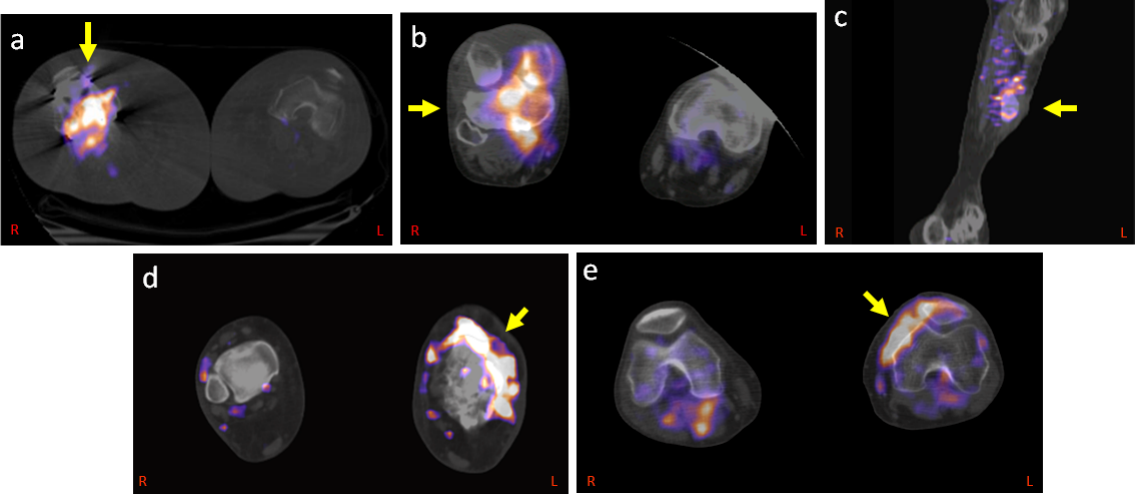
[^124^I]FIAU signal in established infections as imaged by PET/CT: Fused PET and CT images, taken at 2 hours after radiotracer administration, are shown for the following cases. (a) septic arthritis (right knee, Patient 1), (b) septic arthritis (right knee, Patient 4), (c) osteomyelitis (left distal tibia, Patient 5), (d) cellulitis (left lower extremity, Patient 6), (e) necrotizing septic arthritis (left knee, Patient 8).

*Patient 1* had a history of non-insulin dependent diabetes and a right total knee replacement, presented with a painful and swollen right knee. Synovial fluid aspirated from the replaced knee prior to imaging revealed a white blood cell count (WBC) of 11,700 cells/dL with cultures that were positive for methicillin-sensitive *Staphylococcus aureus (*MSSA*)*. A C-reactive protein (CRP) level was elevated at 55 mg/dL (normal value <0.5 mg/dL). [^124^I]FIAU PET/CT imaging at 2 and 24 hours showed intense signal within the right knee (**figure 1a**). The patient underwent surgery and a grossly purulent prosthetic joint was removed and a spacer was placed. Cultures from surgery revealed methicillin-resistant *Staphylococcus aureus* (MRSA).

*Patient 2* suffered from a right hip replacement that had been recurrently infected, requiring multiple revision surgeries. The patient had a normal peripheral WBC count and a CRP value of 8.9 mg/dL. At the time of presentation they had erythema and pain overlying the right hip joint, which was presumed to be a recurrent infection. Her [^124^I]FIAU PET/CT scan was negative. At the time of imaging, the patient was scheduled for surgery. However, it was later decided by the attending orthopaedic surgeon that the overlying erythema was most likely secondary to an abrasion from a brace that the patient wore. Symptoms spontaneously resolved without surgery or antibiotics.

*Patient 3* presented with a left total hip prosthesis that was painful and warm. CRP was 80.1 mg/dL. The [^124^I]FIAU PET/CT images showed a positive signal at the junction of the left femoral and acetabular component at 2 and 24 hours. The patient underwent surgical irrigation and debridement and was noted to have multiple areas of infection within the soft tissue that, when cultured, revealed MRSA.

*Patient 4* had a history of chronic MRSA osteomyelitis involving the right knee requiring multiple surgeries. At the time of imaging, the CRP value was 51.0 mg/dL. The [^124^I]FIAU PET/CT scan demonstrated high signal at 2 and 24 hours within the right knee (**figure 1b**). Subsequent cultures from the knee demonstrated persistent MRSA.

*Patient 5* suffered from a painful and infected non-union fracture of the right tibia as the result of a motor vehicle accident. At the time of imaging, the CRP value was 9.5 mg/dL. The [^124^I]FIAU PET/CT scan demonstrated high signal at 2 hours localized to the lateral aspect of the right tibia (**figure 1c**). The patient underwent a right proximal tibial debridement and open reduction internal fixation. Cultures from this necrotic material revealed *Proteus mirabilis* with numerous gram-positive cocci and a few gram-negative bacilli. Subsequent cultures from the knee demonstrated persistent MRSA.

*Patient 6* had a longstanding history of osteomyelitis in her left distal tibia. The CRP value at the time of imaging was 5.3 mg/dL. The [^124^I]FIAU PET/CT scan showed a positive signal at 2 hours in the distal left tibia (**figure 1d**). They subsequently underwent debridement of this lesion with insertion of calcium-phosphate beads impregnated with antibiotics. Culture results turned out to be negative, but biopsy at the time of culture was positive for chronic inflammation consistent with infection.

*Patient 7* had a history of non-insulin dependent diabetes mellitus had been suffering from recurrent venous stasis ulcers of the left lower extremity for a few years following varicose vein stripping. The [^124^I]FIAU PET/CT scan was positive within an ulcer of the left lower extremity. Cultures from this ulcer revealed multiple organisms including *Enterococcus faecalis, Staphylococcus aureus, Pseudomonas aeruginosa and Proteus mirabilis*.

*Patient 8* presented with swollen and painful left knee suspicious for infection. The CRP was 150.0 mg/dL. An intense signal was found within the left knee on [^124^I]FIAU PET/CT imaging (**figure 1e**). They were found to have necrotizing MRSA osteomyelitis of the proximal tibia.

*Patient 9* had a history of osteoarthritis and prior left total knee replacement surgery. They were placed in this study as a normal control and to determine the normal body biodistribution for [^124^I]FIAU. The [^124^I]FIAU PET/CT study was negative in the normal and prosthetic knee despite the presence of documented degenerative arthritis in both of these joints (**figure S2)**.

### Whole body biodistribution

The biodistribution of [^124^I]FIAU in humans has not been described. Jacobs et. al. reported the lack of [^124^I]FIAU penetration through the blood-brain barrier (BBB) in man, but whole body distribution was not determined.[18] We measured standardized uptake values (SUVs) in tissues from patients who underwent [^124^I]FIAU PET/CT imaging when possible (**table 2**). Because of renal excretion of [^124^I]FIAU, the contents of the urinary bladder accounted for the largest fraction of signal. Muscle, liver and kidneys were also locations of significant [^124^I]FIAU accumulation. Lungs and brain exhibited very little signal, while the heart, spine and sacral marrow showed modest uptake. Some uptake in the liver, pancreas, heart, muscle, and brain was expected due to FIAU phosphorylation by high expression levels of TK2, a mitochondrial enzyme more similar to bacterial thymidine kinases than the major cytoplasmic kinase (TK1).[20], [21] Similar results have been described in radiolabeled FIAU studies of experimental animals.[22]

**Table 2:** [^124^I]FIAU Distribution

Tissue	2 Hour			24 Hour		
	n	Mean SUV	SD	n	Mean SUV	SD
Brain	3	0.83	0.30	0	NA	NA
Lung	5	0.77	0.18	1	0.66	NA
Heart	3	1.77	0.27	1	1.12	NA
Liver	5	8.11	2.56	1	2.88	NA
Spleen	4	2.35	0.26	1	1.59	NA
Kidney	6	15.34	10.84	1	4.91	NA
Small Intestine	6	2.36	0.73	2	1.54	0.51
Large Intestine	6	2.10	0.81	2	1.37	0.23
Muscle	7	2.01	0.56	3	1.60	0.32
Bone	7	2.06	0.52	3	1.98	0.59
Bladder	7	51.02	24.29	1	23.01	NA
Blood	4	3.08	0.44	1	1.66	NA

### Safety in humans

FIAU was developed in the 1990's as a potent anti-viral agent with promising results in early clinical trials of patients suffering from chronic hepatitis B infections. This drug had proved to have very serious toxicity, first described in 1995, when multiple study subjects developed severe mitochondrial toxicity that led to fulminant hepatic failure and death.[23] These clinical trials used daily doses over the course of weeks that were thousands of times greater than the dose we used as a one-time injection of radiotracer. Patients in those trials who received FIAU for less than 4 weeks with a cumulative dose of less than 200 mg had no clinical or biochemical evidence of toxicity. We thereby conservatively estimated that the “no-effect dose” of FIAU would be on the order of 0.1 mg/kg per administration. At the specific activities achieved in our radiosyntheses, doses of FIAU 300,000 times less than the no-effect dose were administered to the patients imaged in the current study. Accordingly, we did not expect any toxicity, but as a precaution, only patients with normal liver function who were not taking any potentially hepatotoxic medications were allowed to participate. To confirm the absence of hepatotoxicity, six of the patients had laboratory values evaluated at baseline and at least 4 weeks after radiotracer injection. These were within normal limits in all cases. All subjects tolerated intravenous injections of [^124^I]FIAU without any evidence of any adverse reactions.

## Discussion

Positive cultures, whether of tissue or blood, are the gold standard for the diagnosis of infection. However, such tests are often invasive and do not always accurately reflect the location and extent of the disease. Imaging is noninvasive and unencumbered by sampling error, and can provide sensitive information about infection location that may enable rapid diagnosis, facilitate monitoring and therapy. Unfortunately, traditional imaging techniques for infection are burdened with low specificities because they detect radiographic changes associated with infection but not the infectious agent itself.

FIAU is a nucleoside analog that freely enters and exits cells. When FIAU is phosphorylated by thymidine kinase (TK), it is selectively trapped within cells that express this enzyme. When coupled with a radioisotope, radiolabeled FIAU (rFIAU) can be detected using SPECT or PET to localize TK-expressing cells in humans. FIAU is an excellent substrate for viral TKs, especially from the Epstein-Barr virus (EBV), and has been used to track the expression of viral TKs encoded by gene therapy vectors *in vivo.* [*24*] rFIAU is attractive for such applications because the major human thymidine kinase (TK1) does not efficiently use FIAU as a substrate.

As noted in the *Introduction*, we became interested in the ability of rFIAU to act as a substrate for bacterial TKs as a result of our preclinical experience with anaerobic, tumorolytic bacteria. The current study was designed to test the potential utility of [^124^I]FIAU in musculoskeletal infections. Infections of prosthetic joints can be difficult to differentiate from trauma, prosthetic loosening, or non-infectious inflammatory conditions. Many commonly used imaging approaches fail to achieve adequate diagnostic specificity in this context because they target inflammation rather than infection. Because rFIAU is incorporated into bacteria rather than inflammatory cells, it should be specific for the infectious process.

There have been other attempts to image bacteria directly by methods that employed radiolabeled immunoglobulins, peptides or antibiotics.[5], [6], [25] [^99m^Tc]Ciprofloxacin has been proffered as a selective bacterial imaging agent, taking advantage of differences between bacterial and human DNA gyrase.[26] However, none of these methods have gained widespread clinical use, partly due to problems in sensitivity or specificity or because the agents possess suboptimal pharmacokinetic properties.[27], [28] Thus, there is an urgent need to develop other modalities for imaging bacterial infections in humans.

In this study, eight patients who were imaged by [^124^I]FIAU PET/CT had positive scans prior to microbiologic or pathological confirmation of infection. Our patient population averaged 59 years of age, so that concurrent degenerative arthritis was often present. The clinical diagnosis of osteoarthritis was specifically documented in Patient 9, our uninfected control. The negative imaging result for this patient demonstrated that [^124^I]FIAU PET/CT can discriminate between infections and inflammation; more patients would have to be studied to determine the generality of this distinction. We also demonstrated that infections in septic native joints, in prosthetic joints and within soft tissues can be imaged with [^124^I]FIAU PET/CT. Furthermore, we attempted to correlate these findings with traditional markers of inflammation such as CRP, which varied widely. For example, Patients 4 and 3 had CRP levels of 51 and 80.1, respectively, with positive imaging studies. Patient 6 had a positive imaging study with a CRP level of only 5.3, attesting to the limited extent to which traditional blood tests can be used to assess infection.

Gram-positive organisms are the most common causes of periprosthetic or joint and bone infections.[29] In the present study, various gram-positive (*staphylococci sp, enterococci sp*), as well as gram-negative (*proteus sp, E.coli sp, pseudomonas sp*) organisms were identified. Interestingly, in one patient (Patient 6) with proven chronic infection no organism was cultured but the FIAU test was clearly positive. This may portend the clinical importance and utility of this new diagnostic test.

However, the current study clearly has several limitations. First, though all patients with infections were identified, this is a pilot study and we have no information as to the sensitivity or specificity of rFIAU imaging in infection. We also do not know how this approach may compare to other modalities, such as MR imaging or the current radiopharmaceutical-based methods of gallium or tagged WBC scanning. We suspect, however, that the rFIAU technique will be more sensitive as there should not be confounding signal from sterile inflammation. Second, we tested only one patient with a non-infectious inflammatory process as a control. Third, the biodistribution analyses showed some radiotracer accumulation within visceral organs, which will likely limit its sensitivity for infections in these regions. Conversely, there was minimal uptake in lungs and brain, making these very attractive sites for future studies. Bilaterally equivalent anatomic organs (such as those of the limbs) were found particularly suitable for [^124^I]FIAU imaging because the contralateral site provides an excellent internal control.

One of the unexpected observations made in the current study was that high signal-to-noise ratios were achieved within two hours of [^124^I]FIAU in patients. This is in contrast to studies with [^124^I]FIAU in mice, which required 16 to 24 hours to obtain optimal images.[10] The difference is likely due to the much greater size of humans compared to mice, permitting easier distinction between localized sites of infection and other sources of signal. In addition to this ability to image rapidly, [^124^I]FIAU can be used soon after surgery, as demonstrated in Patient 6. In contrast, [^99m^Tc]Ciprofloxacin tended to provide false positive studies if imaging was performed within 6 months of surgery.[30], [31] [^99m^Tc]Ciprofloxacin also was associated with high radiopharmaceutical uptake in sterile inflammatory or degenerative conditions.[27] The [^124^I]FIAU preparation and imaging protocol are much simpler than those for the current clinical standard, ^99m^Tc-tagged leukocytes. Tagged leukocyte scans must often be coupled with a bone marrow imaging study to generate acceptable specificity.[32] Despite these potential advantages of [^124^I]FIAU, larger studies will be required to demonstrate its true sensitivity and specificity and its relative strengths and weaknesses over other imaging modalities. [^124^I]FIAU has recently become commercially available and therefore should be relatively simple to test as an imaging agent for various infections in the US.

In summary, we present a new method to image bacterial infection in human subjects. Although we have focused on musculoskeletal bacterial infections in the current pilot study, efforts to extend this technique to infections in other anatomical sites and other organisms that possess a suitable thymidine kinase are underway. Targeting microorganismal thymidine kinase with radiolabeled nucleoside analogs such as FIAU provides a unique approach to the non-invasive diagnosis and therapeutic monitoring of infection.

## Supporting Information

Figure S1 (A) The chemical structure of [^124^I]FIAU. (B) HPLC chromatogram, simultaneously monitored by A254 spectroscopy and gamma counting.
(0.15 MB PPT)

Figure S2 [^124^I]FIAU signal in a healthy subject without infection as imaged by PET/CT: Fused PET/CT images 2 hours after [^124^I]FIAU injection in the axial and coronal views. The patient was a healthy control with a left knee prosthesis. No significant signal was noted in either knee.
(0.30 MB PPT)
