# Supplementary figures and images for: Imaging of Musculoskeletal Bacterial Infections by [124I]FIAU-PET/CT
Source: PLoS One. 2007 Oct 10;2(10):e1007. doi: 10.1371/journal.pone.0001007 (PMC1994593; doi:10.1371/journal.pone.0001007)

## Slide 1
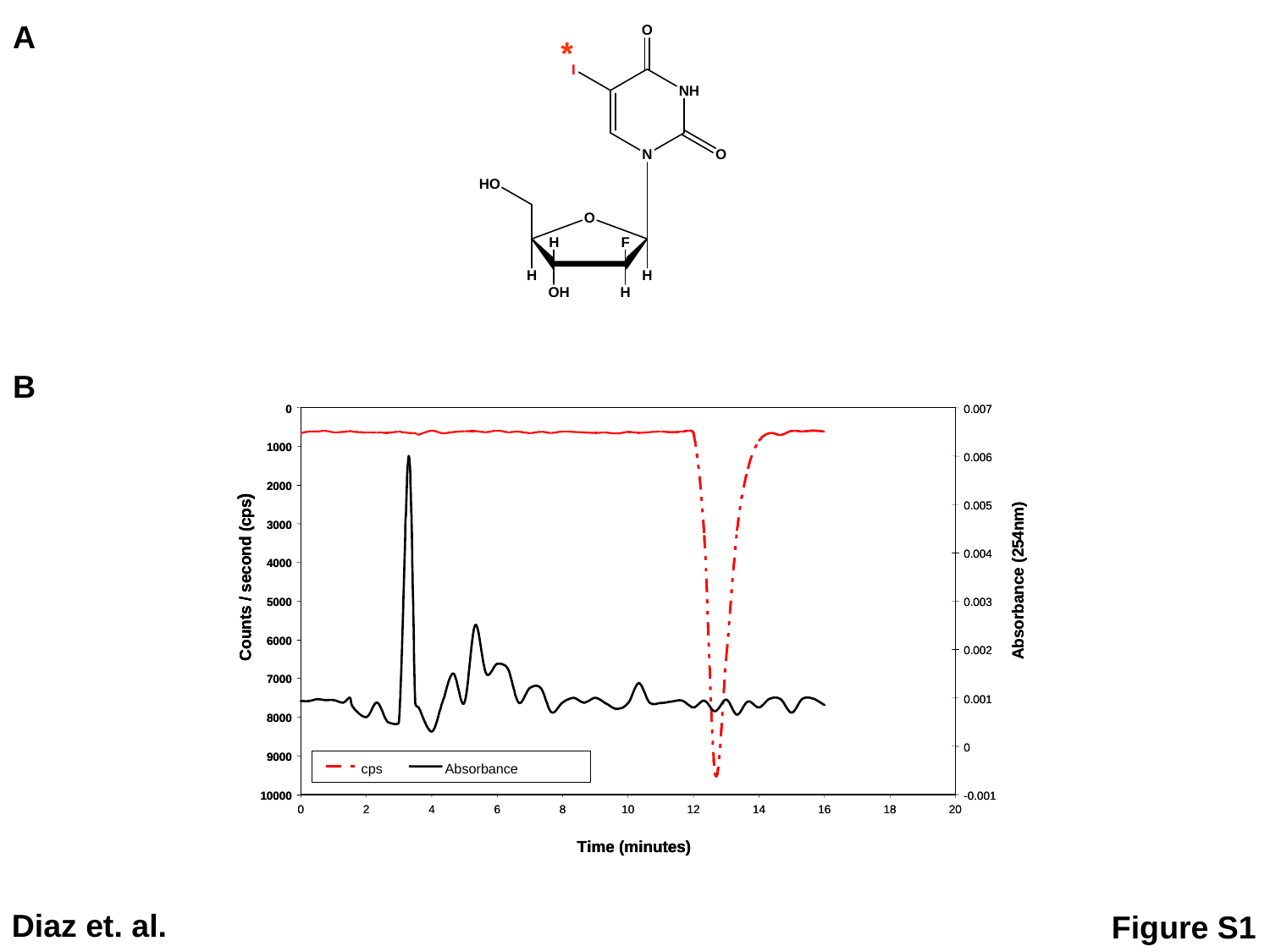

A
*
B
Diaz et. al.
Figure S1

Supplement: Figure S1 — (A) The chemical structure of [124I]FIAU. (B) HPLC chromatogram, simultaneously monitored by A254 spectroscopy and gamma counting. (0.15 MB PPT) [file pone.0001007.s001.ppt]

## Slide 1
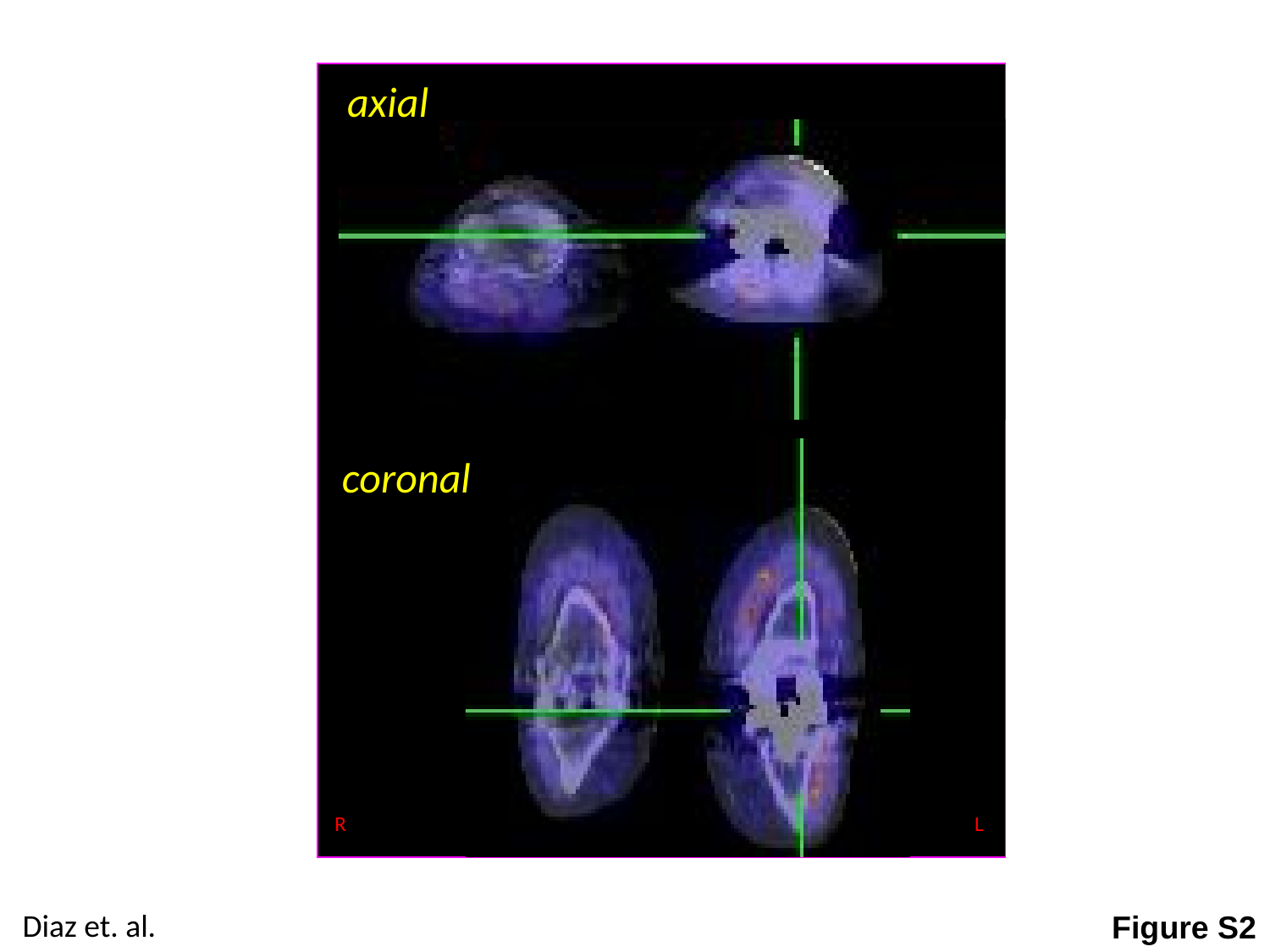

axial
coronal
 R 				 L
Diaz et. al.
Figure S2

Supplement: Figure S2 — [124I]FIAU signal in a healthy subject without infection as imaged by PET/CT: Fused PET/CT images 2 hours after [124I]FIAU injection in the axial and coronal views. The patient was a healthy control with a left knee prosthesis. No significant signal was noted in either knee. (0.30 MB PPT) [file pone.0001007.s002.ppt]
